# Einflussfaktoren auf das Ergebnis der CI-Versorgung: Welche Rolle spielen Ausbildungsabschluss und Berufsausbildung?

**DOI:** 10.1007/s00106-025-01646-9

**Published:** 2025-07-31

**Authors:** Christoph Broeder, Uwe Baumann

**Affiliations:** Klinik für HNO-Heilkunde, Goethe-Universität Frankfurt, Universitätsmedizin, Theodor-Stern-Kai 7, 14, 60590 Frankfurt am Main, Deutschland

**Keywords:** Bildungsgrad, Schulabschluss, Kognition, Freiburger Einsilber, Oldenburger Satztest, Educational attainment, School degree, Cognition, Freiburg monosyllables, Oldenburg sentence test

## Abstract

**Hintergrund:**

Individuelle kognitive Fähigkeiten werden in zunehmendem Maße als ein möglicher Faktor diskutiert, der das Ergebnis der Cochleaimplantat(CI)-Versorgung beeinflussen kann. Vor diesem Hintergrund wurde in der vorliegenden Studie ein möglicher Zusammenhang zwischen dem Schulabschluss und der Berufsausbildung einer größeren Gruppe von CI-Trägern und dem Sprachverstehen untersucht. Als weitere Variablen mit möglichem Einfluss auf das Versorgungsergebnis wurden das Alter bei Implantation, die Dauer der Hörminderung und der Versorgungsmodus einbezogen.

**Methodik:**

Insgesamt wurden *n* = 326 Patientendaten aus der Audiologie-Datenbank der HNO-Universitätsklinik Frankfurt in die Studie einbezogen. Bei diesen Patienten wurde die schulische und berufliche Laufbahn im Rahmen der Anamnese mittels Fragebogen erhoben. Das Sprachverstehen wurde mit dem Freiburger Einsilbertest (FBE) und dem Oldenburger-Satztest im Störgeräusch (OLSA) nach 6 und 12 Monaten bestimmt und mit verschiedenen biografischen und audiologischen Einflussfaktoren korreliert.

**Ergebnis:**

Fälle mit einem höheren Ausbildungsabschluss wiesen 6 und 12 Monate postoperativ ein besseres Ergebnis im FBE 6 M (ANOVA_Welch_; *F* (2, 104) = 5,60; *p* = 0,05) und FBE 12 M (*F* (2, 223) = 3,07; *p* = 0,05; *η*^*2*^ = 0,03) auf als Fälle mit einem beruflich-schulischen Abschluss. Auch beim Sprachverstehen im Störgeräusch (OLSA) zeigte sich 12 Monate postoperativ ein signifikanter Gruppenunterschied. Fälle ohne Abschluss/mit sonstigem Abschluss erzielten schlechtere Testergebnisse als solche mit einem beruflich-schulischen oder einem höheren Abschluss (*F* (2, 74) = 4,41; *p* = 0,02; *η*^*2*^ = 0,11). Andere signifikante Einflussfaktoren auf das Sprachverstehen waren das Alter bei der Implantation und der Versorgungsmodus.

**Schlussfolgerung:**

Die Korrelation zwischen dem schulischen und beruflichen Werdegang und dem Sprachverständnis nach CI-Versorgung war nicht eindeutig. Lediglich der Ausbildungsabschluss zeigte einen signifikanten Zusammenhang mit dem Sprachverstehen. Weitere Forschung ist erforderlich, um einen möglichen Einfluss des Bildungsgrades auf das Sprachverstehen zu bestätigen.

**Zusatzmaterial online:**

Die Online-Version dieses Beitrags (10.1007/s00106-025-01646-9) enthält weiterführende Informationen.

## Abstract (en)

**Background:**

Individual cognitive abilities are increasingly discussed as a potential factor influencing the outcomes of cochlear implant (CI) treatment. In this context, the present study investigated a possible correlation between the secondary and post-secondary education of a large cohort of CI recipients and their speech comprehension. Other variables with a potential influence on the treatment outcome included age at implantation, the duration of hearing loss, and the treatment mode.

**Methods:**

The study included data of *n* = 326 patients from the audiology database of the Frankfurt University ENT Clinic. Secondary and post-secondary education histories were collected as part of the anamnesis using a questionnaire. Speech comprehension was assessed using the Freiburg monosyllabic test (FBE) and the Oldenburg sentence test in noise (OLSA) at 6 and 12 months after implantation and correlated with various biographical and audiological factors.

**Results:**

Patients with higher educational qualifications showed better outcomes in the FBE at 6 months (ANOVA_Welch_; *F* (2, 104) = 5.60; *p* = 0.05) and at 12 months (*F* (2, 223) = 3.07; *p* = 0.05; *η*^*2*^ = 0.03) compared to those with vocational qualifications. In the speech comprehension in noise (OLSA), a significant group difference was observed 12 months postoperatively. Patients without qualifications or with other qualifications scored lower than those with vocational or higher qualifications (*F* (2, 74) = 4.41; *p* = 0.02; *η*^*2*^ = 0.11). Other significant factors influencing speech comprehension included the age at implantation and mode of care.

**Conclusion:**

The correlation between secondary and post-secondary education and speech comprehension after CI treatment was not unequivocal. Only the choice of post-secondary education showed a significant correlation with speech comprehension. Further research is required to confirm a potential relationship.

In der aktuellen Literatur bleibt ein großer Teil der Varianz des Sprachverstehens nach Cochleaimplantat(CI)-Versorgung ungeklärt. Individuelle kognitive Fähigkeiten sind derzeit Gegenstand einer kontroversen Diskussion als möglicher Einflussfaktor auf das CI-Ergebnis. Einen Teil dieser Varianz könnte der Bildungsgrad als möglicher Indikator für kognitive Fähigkeiten der Patienten erklären. Bisherige Studien konnten keine signifikante Korrelation des Bildungsgrades mit dem Sprachverstehen zeigen, weisen aber auf weiteren Forschungsbedarf hin.

Das Cochleaimplantat (CI) ist eine Hörprothese für Gehörlose sowie hochgradig Schwerhörige, bei denen ein Hörgerät keine ausreichende Versorgung mehr darstellt. Es zählt als Standardtherapie für Patienten mit starkem Hörverlust [21, 26]. Wie von Lenarz et al. beschrieben wurde, ist das CI-Ergebnis sehr individuell und hängt von vielen Faktoren ab. Ätiologie, Dauer und Beginn der Hörminderung sowie das Alter bei Implantation sind in der Literatur gut dokumentierte Einflussfaktoren [1, 3, 8, 22]. Das CI fördert nicht nur ein besseres Sprachverstehen und damit eine bessere auditive Wahrnehmung, sondern erhöht ebenso die Lebensqualität der Anwender [36, 39]. Eine ständige Weiterentwicklung sowohl der Implantate, CI-Prozessoren, Elektrodenbauformen und der verwendeten Software als auch der klinischen Bedingungen der Patientenversorgung könnten zu einer Veränderung des Einflusses dieser Faktoren auf das Sprachverstehen führen. So zeigt Blamey et al. in 2 multizentrischen Studien (1996, 2013) eine Abnahme der Bedeutung der biografischen und audiologischen Einflussfaktoren hinsichtlich des Sprachverstehens. Diese erklären 2013 nur noch 10 % der Varianz im Vergleich zu 21 % im Jahr 1996 [2, 3]. Auch neuere Studien setzen sich mit der Relevanz bereits weitläufig etablierter Einflussfaktoren auseinander. In seiner Metanalyse zeigt Zhao et al. 2020, dass die präoperativ gemessenen patientenspezifischen Faktoren zwar signifikanten Einfluss auf das Sprachverstehen aufweisen, jedoch kumuliert nur knapp 10 % der Varianz erklären und damit nur limitiert Unterstützung im Rahmen der klinischen Entscheidungen bei CI-Versorgungen bieten können [41]. Die Vorhersagbarkeit des CI-Erfolges und die damit verbundene Planbarkeit der Therapie sind insgesamt immer noch sehr limitiert. Studien deuten darauf hin, dass ältere Menschen mit Hörverlust im Vergleich zu jüngeren Erwachsenen trotz ähnlicher Ergebnisse in der Reintonaudiometrie ein schlechteres Sprachverstehen aufweisen [6, 40]. Dies könnte unter anderem mit der altersbedingten Abnahme kognitiver Funktionen wie Verarbeitungsgeschwindigkeit, Arbeitsgedächtnis und selektiver Aufmerksamkeit zusammenhängen. In ruhiger Umgebung erfolgt die Verarbeitung von Sprache nahezu automatisch, während in lauter Umgebung, vor allem bei einem beeinträchtigten Gehör, die eingehenden neuronalen Aktivitätsmuster verzerrt werden und der Abgleich mit gespeicherten Repräsentationen möglicherweise nicht mehr eindeutig ist [27]. Diese Verzerrungen erfordern kognitive Ressourcen beim Sprachverstehen, was zu Problemen bei denjenigen mit einer Reduktion der kognitiven Verarbeitungsgeschwindigkeit führen kann. Das Modell der Sprachverarbeitung zeigt, dass ein Großteil der Varianz im Sprachverstehen bei älteren Personen mit Unterschieden in deren kognitiver Leistungsfähigkeit begründet ist [12]. Kognition ist definiert als ein interner Prozess, der daran beteiligt ist, die Umgebung zu verstehen und zu entscheiden, welche Handlung angemessen sein könnte [9]. Auch Heydebrand et al. und Moberly et al. untersuchen 2007 beziehungsweise 2018 kognitive Fähigkeiten wie beispielsweise das auditive Arbeitsgedächtnis als mögliche Einflussfaktoren auf das CI-Ergebnis mit disparaten Ergebnissen [19, 32]. Heydebrand et al. berichten bei *n* = 37 Studienteilnehmern über keine Korrelation zwischen kognitiven Parametern und dem CI-Ergebnis 6 Monate postoperativ [19]. Im Gegensatz dazu zeigen Moberly et al., dass bei *n* = 31 Patienten die Ergebnisse des Sprachverstehens mit dem auditiven Arbeitsgedächtnis korrelieren [32]. Beide Autoren kommen zur Aussage, dass weiterer Forschungsbedarf besteht, um den Einfluss kognitiver Effekte auf das Sprachverstehen zu bestimmen.

In der vorliegenden Studie wird der Bildungsgrad der Patienten als Indikator für ihre kognitiven Fähigkeiten untersucht. Der Bildungsgrad umfasst die Art der besuchten Einrichtung und der von den Patienten höchste erreichte Abschluss. Zahlreiche Studien belegen, dass bei Normalhörenden ein höherer Bildungsgrad mit einer besseren kognitiven Leistungsfähigkeit korreliert [7, 30, 33]. In dem Review von Lövdén et al. zeigen die Autoren, dass in der aktuellen Literatur ein positiver Einfluss des Bildungsgrades auf die Kognition beschrieben ist. Chen et al. berichten ebenfalls bei *n* = 659 normalhörenden Senioren von einem signifikanten Zusammenhang zwischen einem hohen Bildungsgrad und einem hohen Maß an umfassender kognitiver Leistungsfähigkeit. Außerdem ist bei diesen Patienten eine verzögerte altersbedingte Reduktion der Kognition zu beobachten [7]. Es lässt sich annehmen, dass die beschriebenen Auswirkungen des Bildungsgrades auf die Kognition auch bei hörgeminderten Patienten bestehen. Dementsprechend ist es möglich, den Bildungsgrad als einen Indikator für die Kognition der Patienten heranzuziehen. Die Relevanz des Bildungsgrades im Zusammenhang mit dem Sprachverstehen nach CI-Versorgung ist in der Literatur bisher noch wenig untersucht. Heutink et al. und Lazard et al. untersuchten unter anderem den Einfluss des Bildungsgrades auf das Sprachverstehen bei Kollektiven von *n* = 129 und *n* = 2251 CI-Probanden [18, 25]. Beide Arbeitsgruppen konnten keinen signifikanten Zusammenhang mit dem CI-Ergebnis feststellen. Unter Umständen waren die gewählten Gruppengrößen zu klein oder die Gruppierung der Bildungsstufen selbst zu unscharf, sodass möglicherweise vorhandene signifikante Korrelationen überdeckt werden.

Vor diesem Hintergrund rückt die Definition des CI-Erfolgs als zentrales Kriterium für die Beurteilung möglicher Zusammenhänge in den Fokus. Das CI-Ergebnis beziehungsweise der CI-Erfolg ist in dieser Studie definiert als das Sprachverstehen nach CI-Versorgung, gemessen mittels der Sprachverstehenstests Freiburger Einsilbertest (FBE) und Oldenburger Satztest (OLSA). Unberücksichtigt bleiben weitere Faktoren wie z. B. soziale Teilhabe, Lebensqualität und Kommunikation, welche in der Regel ein Teil des CI-Ergebnisses darstellen. Ziel der vorliegenden Studie ist die Untersuchung der Fragestellung, ob der Bildungsgrad der Patienten mit dem Sprachverstehen nach CI-Versorgung korreliert.

## Material und Methoden

### Datenerhebung

Die im Rahmen der vorliegenden Studie durchgeführte retrospektive Datenerhebung erfasste Daten von Patienten, die im Zeitraum Oktober 2008 bis Oktober 2021 eine Hörimplantat-Versorgung erhielten. Die Datensätze wurden aus der Audiologie-Datenbank der HNO-Universitätsklinik Frankfurt entnommen (ENT-Statistics Version: 4.1.522.546, Fa. Innoforce, Liechtenstein). Im Rahmen der Anamneseerhebung bzw. -aktualisierung wurden die im genannten Intervall versorgten Patienten im Zeitraum zwischen 2017 und 2021 gebeten, Angaben zu ihrem schulischen und beruflichen Werdegang zu tätigen. Die Angaben wurden in ein Formular der Datenbank („Arbeit und Leben“) übertragen. Die Datenbankabfrage generierte anschließend eine Excel-Tabelle (Fa. Microsoft Corporation, Redmond, WA, USA, Version: 16.74) mit Patientenstammdaten, Behandlungsdaten, Daten zur Patientenhistorie, Art der Versorgung, Angaben zu Lebensumständen und Angaben aus dem „Arbeit und Leben“-Formular. Die Datenbankabfrage über den Zeitraum von Oktober 2008 bis Oktober 2021 ergab *n* = 821 Einträge zu Patientenversorgungen mit CI, Bonebridge (Fa. Med-EL, Innsbruck, Österreich)- oder Vibrant Soundbridge Implantaten (VSB, Fa. Med-EL, Innsbruck, Österreich). Es lagen für diese Versorgungen insgesamt *n* = 609 Fragebögen zum Bildungsgrad („Arbeit und Leben“-Formular) vor. Nach Anwendung der zusätzlichen Einschlusskriterien – Deutsch als Muttersprache (*n* = 235 Drop-outs mit anderer Muttersprache) und Angaben zum Versorgungsmodus (*n* = 23 Drop-outs mit fehlenden Angaben zum Versorgungsmodus) – reduzierte sich die Studienpopulation auf *n* = 351 verbleibende Fälle. Für *n* = 120 Fälle mit bilateraler CI-Versorgung wurden die Daten derjenigen CI-Seite in die Studie integriert, die postoperativ ein besseres Ergebnis des Freiburger Einsilbertests sowie niedrigere Werte des Signal-Rausch-Verhältnisses („signal-to-noise ratio“, SNR) als Ergebnis des Oldenburger Satztests (OLSA) aufwiesen. Bei disparaten Testresultaten wurde das durchschnittliche Testergebnis anhand der betrachteten Nachkontrollzeitpunkte (6 und 12 Monate postoperativ) gebildet und die bessere Seite bestimmt. Dies verhinderte, dass bilateral CI-versorgte Patienten doppelte Datensätze zu den Angaben des Bildungsgrades erzeugten, was zu einer Verzerrung der Ergebnisse geführt hätte. Darüber hinaus wurden Fälle ausgeschlossen, für die keine FBE-Testergebnisse vorlagen (*n* = 6) oder bei denen ein Hörnervdefekt diagnostiziert wurde (*n* = 2). Nach Ausschluss von Versorgungen mit Bonebridge- und Vibrant-Soundbridge-Implantaten (*n* = 17) betrug die endgültige Fallzahl *n* = 326.

### Sprachverstehen in Ruhe und im Störgeräusch

Die für die folgenden Auswertungen herangezogene Studienpopulation bestand aus 190 Frauen und 136 Männern (Tab. 1). Der Implantationszeitpunkt lag im Mittel bei 53,0 Jahren (SD = 16,5). Bei bestmöglicher Hörgeräteversorgung lag das präoperativ ermittelte durchschnittliche Testergebnis des FBE bei 16,5 % (SD = 21,9). Nach 6 Monaten postoperativ wurde ein durchschnittlicher FBE-Wert von 57,4 % (SD = 26,1) und nach 12 Monaten postoperativ ein durchschnittlicher FBE-Wert von 63,9 % (SD = 23,0) erreicht. Die Sprachverständlichkeitsschwelle im Störgeräusch (OLSA) verringerte sich zwischen den Testintervallen 6 und 12 Monate um 0,6 dB SNR von L50 = − 0,8 dB SNR (SD = 2,5) auf L50 = −1,4 dB SNR (SD = 2,2). Zur Korrelationsanalyse mit den Ergebnissen des FBE verblieben nach listenweisem Fallausschluss *n* = 226 Fälle; zur Korrelationsanalyse mit den Ergebnissens des OLSA im Störgeräusch *n* = 77 Fälle.

**Tab. 1:** Demografische Daten und Sprachtestergebnisse

	Anzahl* n*	Minimum	Maximum	Mittelwert	Standardabweichung
Alter in Jahren	326	20,0	92,0	58,6	16,0
Alter bei Implantation in Jahren	326	2,2	88,4	53,0	16,5
FBE prä-OP (%)	217	0,0	85,0	16,5	21,9
FBE 6 M post-OP (%)	217	0,0	100,0	57,4	26,1
FBE 12 M post-OP (%)	217	5,0	100,0	63,9	23,0
OLSA 6 M (dB SNR)	77	−5,3	5,0	−0,8	2,5
OLSA 12 M (dB SNR)	77	−6,3	5,0	−1,4	2,2

Ergebnisse des Freiburger Einsilbertests (FBE, 65-dB-SPL-Freifelddarbietung) und des Oldenburger Satztest im Störgeräusch (OLSA, listenweiser Fallausschluss, Störgeräusch OlNoise, Sprachpegel 65 dB SPL fest, Störgeräuschpegel adaptiv, S0N0-Darbietung)

### Alter bei Implantation und Dauer der Hörminderung

Das Durchschnittsalter der Kohorte lag bei *M* = 58,6 Jahren (*SD* = 16,0) mit einer Spanne von 20 bis 92 Jahren (Tab. 1). Die von den Patienten anamnestisch angegebene Dauer der Hörminderung wurde kategorisch erfasst. Die Schwerhörigkeit entwickelte sich überwiegend peri- oder postlingual (*n* = 199), während *n* = 54 Fälle bereits eine kongenitale Schwerhörigkeit aufwiesen und bei *n* = 73 Fällen keine Angaben zur Dauer der Hörminderung vorlagen. Die größte Gruppe bildeten die Patienten, bei welchen innerhalb von 20 Jahren postlingual ihre Schwerhörigkeit auftrat (*n* = 91).

### Versorgungsmodus

Die Gruppierung des Modus erfolgte anhand der Beschreibung der Prüfbedingung im Kommentarfeld des im Freifeld aufgezeichneten Sprachaudiogramms nach CI-Implantation. Der Versorgungsmodus wurde in 5 Kategorien unterteilt. *Unilateral(e)* Versorgung beschrieb Fälle mit ausschließlich einseitiger CI-Nutzung, da aufgrund funktioneller Taubheit das Gegenohr nicht erfolgreich mit Hörgeräten versorgt werden konnte. Bei einem asymmetrischen Hörverlust („asymmetric hearing loss“, *AHL*) bestand neben der CI-Versorgung eine gut unterstützende Hörgeräte-Anpassung am Gegenohr (Testergebnis mit Hörgerät im Freiburger Einsilbertest, FBE, besser als 50 %). Die Kategorie *Bimodal* umfasste ebenfalls CI-Nutzer mit Hörgeräteversorgung am Gegenohr, aber mit weniger Gewinn durch die Hörgeräteversorgung (Testergebnis mit Hörgerät im FBE ≤ 50 %). Fälle mit beidohriger CI-Versorgung wurden der Kategorie *Bilateral* zugerechnet. Bei bilateral CI-versorgten Patienten wurde seitengetrennt geprüft. Zur weiteren Auswertung wurde die CI-Seite mit dem besseren FBE-Ergebnis bestimmt und der Datensatz in die Statistik integriert. Bei Normakusis des Gegenohrs wurde der Fall als *SSD* („single-sided deafness“) eingeordnet. Für alle Gruppen außer *AHL* existierten einige Fälle mit elektrisch-akustischer Stimulation (EAS), welche ihren entsprechenden Gruppen zugeordnet wurden. Es lag folgende Verteilung innerhalb der Kategorien vor: *Unilateral* (*n* = 22), *AHL* (*n* = 38), *Bimodal* (*n* = 87), *Bilateral* (*n* = 120) und *SSD* (*n* = 59).

### Kindergarten, Schulbildung und -abschluss, Berufslaufbahn

Die den Patienten zugehörigen Bildungsinformationen wurden durch einen Fragebogen erfasst („Arbeit und Leben“-Formular, Datenbank ENT-Statistics, Fa. Innoforce, Liechtenstein; s. elektronisches Zusatzmaterial online) und setzten sich aus Angaben zur Betreuung im Kindesalter, zur Schulform und zu den schulischen und beruflichen Abschlüssen zusammen. Die Betreuung im Kindesalter gliederte sich in 4 Gruppen: *kein Kindergarten, Regelkindergarten, Schwerhörigenkindergarten* und *Integrationskindergarten*. Der Unterschied zwischen den letzten beiden Gruppen bestand darin, dass im Schwerhörigenkindergarten ausschließlich hörbeeinträchtigte Kinder betreut wurden im Gegensatz zu einem Integrationskindergarten, bei welchem auch Kinder mit Normakusis anwesend waren.

Die Kategorisierung der von den Patienten besuchten Schuleinrichtungen erfolgte anhand des Betreuungsgrades. Alle Fälle ohne spezielle Förderung für Hörgeschädigte oder Gehörlose wurden der Kategorie *Regelschule *zugeordnet. Unter der Schulform *Gehörlosenschule* wurden Patienten zusammengefasst, die ausschließlich an einer Schule für Gehörlose unterrichtet wurden. Alle anderen Fälle, denen eine Unterstützung in jeglicher Form zukam, wurden unter *Sonstiges* zusammengefasst. Darunter zählten: Schwerhörigenschulen, Schulen für Lernhilfe und Integrationsschulen.

In der Studie erfolgte zudem eine Klassifizierung basierend auf dem Schul- und Ausbildungsabschluss. Der Schulabschluss wurde in folgende Kategorien unterteilt: *Hauptschulabschluss, Realschulabschluss, Abitur* und *kein Abschluss*. Innerhalb der Kategorie *Abitur* wurden auch die Fachhochschulreife und die polytechnische Oberstufe eingeschlossen. Die berufliche Laufbahn wurde in drei Kategorien gegliedert: *kein oder sonstiger Berufsabschluss, beruflich-schulischer Abschluss* und *höherer Abschluss*. Zu letztem zählten Abschlüsse einer Meister- oder Technikerausbildung sowie Fachhochschul- und Universitätsabschlüsse.

### Sprachaudiometrie

Das Sprachverstehen in Ruhe wurde mittels des Freiburger Einsilbertests [15] bei 65 dB SPL Wiedergabepegel im Freifeld (Abstand zum Lautsprecher 1 m) in akustisch isolierten Audiometrieräumen geprüft. Zur Datenerhebung wurden die Ergebnisse der präoperativen Tests (mit Hörgeräteversorgung) sowie die Ergebnisse der CI-Versorgung nach 6 und 12 Monaten herangezogen. Die Tests erfolgten mit den Alltagseinstellungen des CI-Prozessors der Probanden. Zur Prüfung der Verständlichkeit von Sprache unter Störgeräuschbedingungen wurde der Oldenburger Satztest (OLSA) [24], bei einem festen Sprachpegel von 65 dB SPL und adaptivem Rauschpegel eingesetzt (Sprach- und Rauschsignal frontal, *S0N0*-Bedingung). Bei Probanden mit einseitiger Taubheit und nutzbarer Hörfunktion des Gegenohres wurde das kontralaterale Ohr mittels Ohrstöpseln und Kapselgehörschutz (Peltor) doppelt abgeschirmt. Das Ziel des OLSA war die Bestimmung der Sprachverständlichkeitsschwelle (SVS, *„*speech reception threshold“,* SRT*), also dem Verhältnis zwischen Sprachsignal und Störgeräusch, bei welchem der Proband noch 50 % der Sätze korrekt erkannt hat. Das Testergebnis wurde als Signal-Rausch-Verhältnis („signal-to-noise ratio“, SNR) mit der Einheit dB SNR aufgezeichnet. Ein positiver Wert wies auf ein schlechteres Sprachverstehen hin, da bei Erreichen der SVS der Sprachpegel höher war als der Rauschpegel.

### Statistik

Die statistische Analyse der Daten erfolgte mit SPSS Version 28.0 (Fa. IBM Corp., Armonk, NY, USA). Die Analyse der demografischen Daten erfolgte unter Erhebung des Durchschnitts und der Standardabweichung. Die metrisch skalierten Daten zeigten keine Normalverteilung (Shapiro-Wilk-Test). Mögliche Zusammenhänge zwischen den metrischen Variablen und den Ergebnisvariablen (FBE und OLSA) wurden mit dem Sperman´schen-Rangkorrelationskoeffizienten dargestellt. Die Abhängigkeit nominal skalierter Variablen zu den Ergebnisvariablen wurde unter Verwendung der ANOVA überprüft. Bei Varianzen-Inhomogenität, getestet nach Levene, wurde der Zusammenhang mittels Welch-ANOVA bewertet. Um für den Einfluss möglicher Kovariaten zu bereinigen, wurde anschließend eine ANCOVA berechnet. Die Effektstärke und gleichzeitig die Varianz, die durch die ANOVA/ANCOVA erklärt werden kann, wurde mittels Eta-Quadrat/partiellem Eta-Quadrat dargestellt. Zur Prüfung auf mögliche signifikante Unterschiede in den Probandengruppen wurde bei homogenen Varianzen der Post-hoc-Test nach Bonferroni und bei inhomogenen Varianzen der Games-Howell-Test angewandt.

## Ergebnisse

### Korrelations- und Varianzanalyse

Die Ergebnisse der Varianzanalyse und der Korrelationsanalyse unter Verwendung des Spearman´schen-Rangkorrelationskoeffizienten sind in Tab. 2 aufgelistet. Es wurde auf eine prozessor- und elektrodenspezifische Auswertung verzichtet, da diese aufgrund der großen Anzahl unterschiedlicher Gerätetypen und der daraus resultierenden kleinen Gruppengröße kaum aussagekräftige Ergebnisse erbrachte.

**Tab. 2:** Korrelation der Ergebnisvariablen mit den metrischen und kategorischen Variablen unter Verwendung des Spearman´schen-Rangkorrelationskoeffizienten und der Varianzanalyse (ANOVA)

	Spearman-Rho *r*_*S*_* | p*			
Metrische Variablen	FBE 6 M†	FBE 12 M†	OLSA 6 M††	OLSA 12 M††
Alter bei Implantation	−0,09 | 0,17	*−0,26 | <* *0,001*	0,21 | 0,07	*0,25 | 0,03*
Kategorische Variablen	ANOVA-Signifikanz			
Dauer Hörminderung	0,10	0,19	0,33	0,20
Modus	*<* *0,001*	*<* *0,001*	0,07	*<* *0,01*
*Bildungsgrad*				
Kindergartenform	0,42	0,12	0,38	0,29
Schulform	0,07	0,05	0,59	0,43
Schulabschluss	0,32	0,37	0,17	0,14
Ausbildungsabschluss	*<* *0,01*	*0,05*	0,10	*0,02*

*n* (†) = 226, *n* (††) = 77, Freiburger Einsilber (FBE, 65-dB-SPL-Freifelddarbietung) und Oldenburger Satztest im Störgeräusch (OLSA, listenweiser Fallausschluss, Störgeräusch OlNoise, Sprachpegel 65 dB SPL fest, Störgeräuschpegel adaptiv, S0N0-Darbietung), signifikante Ergebnisse hervorgehoben.

### Alter bei Implantation und Dauer der Hörminderung

Es bestand ein signifikanter Zusammenhang zwischen dem Alter bei Implantation und dem FBE-Testergebnis 12 Monate postoperativ: *r*_*S*_ (224) = −0,26; *p* < 0,001. Das negative Vorzeichen des Korrelationskoeffizienten zeigte dabei, dass bei zunehmendem Alter das Ergebnis des FBE abnahm. Eine signifikante Korrelation war auch hinsichtlich des OLSA-Testergebnisses 12 Monate postoperativ festzustellen: *r*_*S*_ (75) = 0,25; *p* = 0,03. Mit zunehmendem Alter zeigte sich ein Ansteigen des SNR-Wertes, gekennzeichnet durch einen positiven Korrelationskoeffizienten. Die Dauer der Hörminderung zeigte keine Korrelation zu den Sprachverstehenstests (Tab. 2).

### Versorgungsmodus

**Abb. 1:**
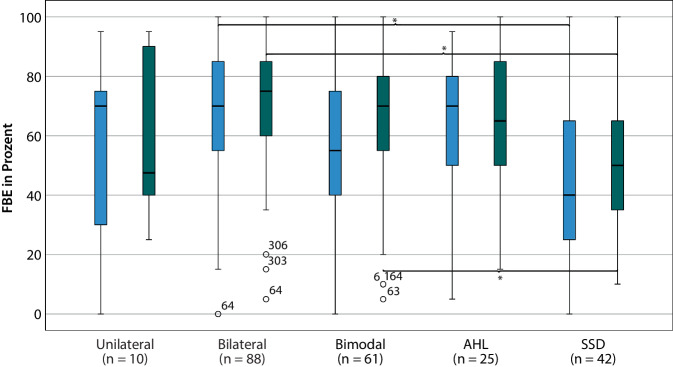
Boxplot FBE 6 M (*blau*) und 12 M post-OP (*grün*) im Zusammenhang mit dem Versorgungsmodus, *n* = 226, (bessere Seite bei bilateral versorgten Patienten), *p < 0,05; FBE (65-dB-SPL-Freifelddarbietung). *AHL* asymmetrischer Hörverlust, („asymmetric hearing loss“), *SSD* einseitiger Hörverlust („single-sided deafness“), *FBE* Freiburger Einsilbertest

Zwischen den Versorgungsmodi gab es signifikante Unterschiede in Bezug auf das Ergebnis des FBE sowohl 6 Monate (*F* (4, 221) = 5,82; *p* < 0,001; *η*^*2*^ = 0,10), als auch 12 Monate (*F* (4, 221) = 6,95; *p* < 0,001; *η*^*2*^ = 0,11) postoperativ; im Störgeräusch nur beim Testintervall 12 Monate (*F* (4, 72) = 4,10; *p* < 0,01; *η*^*2*^ = 0,18). Die Post-hoc-Analyse zeigte, dass Fälle mit einer SSD-Versorgung zu beiden Testzeitpunkten schlechtere Testergebnisse im FBE aufwiesen als solche mit bilateralem Versorgungsmodus (*p* < 0,001; Tab. 3). Nach 12 Monaten zeigten SSD-Fälle auch ein schlechteres Ergebnis im FBE im Vergleich zu den bimodal versorgten Fällen (*p* = 0,03; Abb. 1). Die OLSA-Ergebnisse (nicht dargestellt) zeigten 12 Monate postoperativ einen signifikant negativen Zusammenhang zwischen SSD-Versorgungen und den Gruppen: *Bilateral* (*p* < 0,01), *Bimodal* (*p* = 0,03) und *AHL* (*p* = 0,04). SSD-Fälle wiesen damit ein besseres Sprachverstehen auf als die anderen Versorgungsart-Gruppen.

**Tab. 3:** Signifikante Post-hoc-Vergleiche nach Bonferroni-Korrektur

Abhängige Variable	Testvariable	(I) Modi	(J) Modi	Mittelwertdifferenz (I–J)	Standardfehler	Signifikanz
FBE 6 Monate	Modus	SSD	Bilateral	−20,7	4,7	*<* *0,001*
*	Ausbildungsabschluss	Berufl.-schulischer Abschluss	Höherer Abschluss	−12,4	3,7	*<* *0,01*
FBE 12 Monate	Modus	SSD	Bilateral	−21,4	4,1	*<* *0,001*
		SSD	Bimodal	−13,3	4,4	*0,03*
	Ausbildungsabschluss	Berufl.-schulischer Abschluss	Höherer Abschluss	−8,3	3,4	*0,04*
OLSA 12 Monate	Modus	SSD	Bilateral	−2,5	0,7	*<* *0,01*
		SSD	Bimodal	−2,1	0,7	*0,03*
		SSD	AHL	−2,4	0,8	*0,04*
	Ausbildungsabschluss	Kein Abschluss/ sonstiger	Berufl.-schulischer Abschluss	2,1	0,7	*0,02*
		Kein Abschluss/ sonstiger	Höherer Abschluss	1,8	0,7	*0,04*

*n* (FBE) = 226, *n* (OLSA) = 77, Freiburger Einsilber (FBE, 65-dB-SPL-Freifelddarbietung) und Oldenburger Satztest im Störgeräusch (OLSA, listenweiser Fallausschluss, Störgeräusch OlNoise, Sprachpegel 65 dB SPL fest, Störgeräuschpegel adaptiv, S0N0-Darbietung), * = Post-hoc-Test nach Games-Howell, da keine Varianzenhomogenität bestand.

### Kindergarten, Schulbildung und -abschluss, Berufslaufbahn

Bei einem Großteil der Patienten begann der Bildungsweg mit dem Besuch eines Regelkindergartens (*n* = 200), während *n* = 25 eine besondere Förderung zuteil kam, entweder in Form eines Schwerhörigenkindergartens (*n* = 19) oder eines Integrationskindergartens (*n* = 6). *n* = 101 nahmen keine Betreuung in einem Kindergarten in Anspruch.

**Abb. 2:**
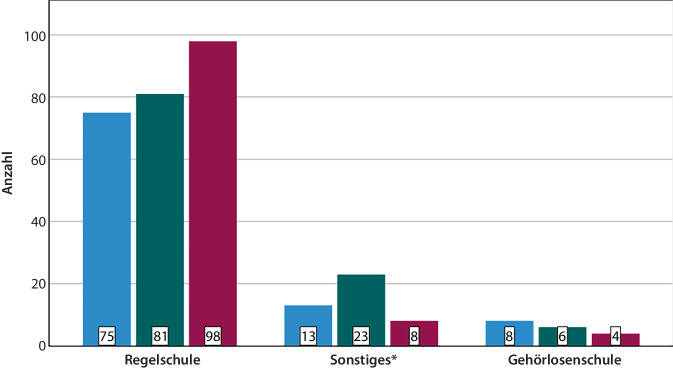
Balkendiagramm zum Schulabschluss abhängig von der Schulform, *blau* = Hauptschulabschluss, *grün* = Realschulabschluss, *rot* = Abitur, Gesamtzahl *n* = 316, 10 Fälle ohne Schulabschluss, *Asterisk* Einrichtungen für Lernhilfe (*n* = 6), Integrationsschulen (*n* = 6) und Schwerhörigenschulen (*n* = 36)

Der überwiegende Teil der Patienten besuchte eine Regelschule (*n* = 260). Die meisten Patienten erlangten dort als Abschluss die allgemeine Hochschulreife (*n* = 98), gefolgt vom Realschulabschluss (*n* = 81) und Hauptschulabschluss *(n* = 75). *n* = 6 Fälle schlossen ihre schulische Laufbahn ohne einen Abschluss ab (Abb. 2). Unabhängig von der Schulform erlangten *n* = 110 Patienten das Abitur, *n* = 110 einen Realschulabschluss, *n* = 96 einen Hauptschulabschluss, und *n* = 10 blieben ohne Abschluss. Zum Abschluss *Abitur* zählten auch die Fachhochschulreife (*n* = 38) und der Abschluss der polytechnischen Oberstufe (*n* = 7). Die Gruppe der *Sonstigen* Schulen umfasste Einrichtungen für Lernhilfe (*n* = 6), Integrationsschulen (*n* = 6) und Schwerhörigenschulen (*n* = 36).

**Abb. 3:**
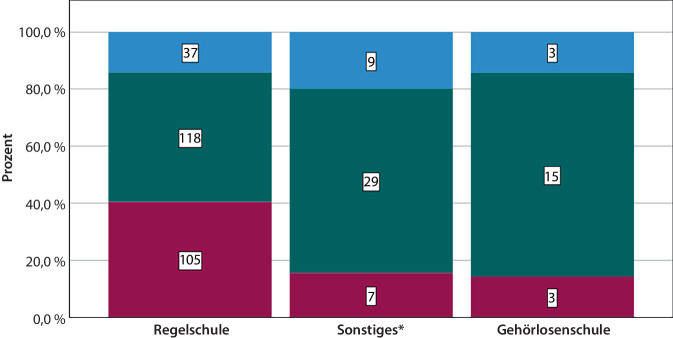
Balkendiagramm zum Ausbildungsabschluss abhängig von der Schulform, *blau* = kein Abschluss/Sonstiger, *grün* = berufl.-schulischer Abschluss, *rot* = höherer Abschluss, Gesamtzahl *n* = 326, *Asterisk* Einrichtungen für Lernhilfe (*n* = 6), Integrationsschulen (*n* = 6) und Schwerhörigenschulen (*n* = 36)

Nach der Schulzeit absolvierte der Großteil der Patienten eine berufliche oder schulische Ausbildung (*n* = 162), während einige eine Meister- oder Technikerschule besuchten (*n* = 43) oder einen Fachhochschul- oder Universitätsabschluss erlangten (*n* = 72). Keinen oder einen anders gearteten beruflichen Abschluss erreichten *n* = 49 Patienten (Abb. 3).

Hinsichtlich des Bildungsgrades haben weder Kindergartenform (FBE 12 M: (*F* (3, 222) = 1,97; *p* = 0,12), OLSA 12 M: (*F* (3, 73) = 1,28; *p* = 0,29)) noch Schulabschluss (FBE 12 M: (*F* (3, 222) = 1,06; *p* = 0,37), OLSA 12 M: (*F* (3, 73) = 1,86; *p* = 0,14)) einen Einfluss auf die Ergebnisse des Sprachverstehens in Ruhe oder im Störgeräusch. Im Hinblick auf die Variable „Schulform“ bestand 12 Monate postoperativ ein dahingehender Trend, dass Patienten, welche eine Gehörlosenschule besuchten, ein etwas schlechteres Sprachverstehen in Ruhe zeigten (FBE 12 M: (*F* (2, 223) = 3,01; *p* = 0,05)), als Patienten welche an einer Regelschule (*p* = 0,08) oder sonstigen Schule unterrichtet wurden (*p* = 0,06).

**Abb. 4:**
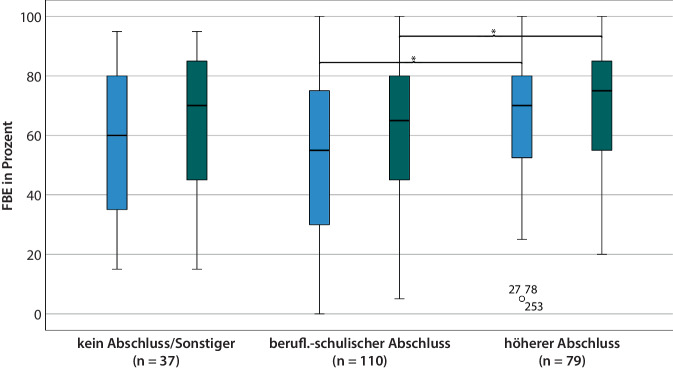
Boxplot FBE-Testergebnisse 6 M (*blau*) und 12 M (*grün*) post-OP im Zusammenhang mit dem Ausbildungsabschluss, *n* = 226, *p < 0,05; FBE (65-dB-SPL-Freifelddarbietung). *FBE* Freiburger Einsilbertest

Für die Variable „Ausbildungsabschluss“ bestand ein signifikanter Gruppenunterschied (FBE 6 M: ANOVA_Welch_ (*F* (2, 104) = 5,60; *p* = 0,05), FBE 12 M: (*F* (2, 223) = 3,07; *p* = 0,05; *η*^*2*^ = 0,03)). Fälle mit beruflich-schulischem Abschluss erreichten signifikant schwächere FBE-Test-Ergebnisse als solche mit einem höheren Abschluss, sowohl 6 Monate (*p* < 0,01) als auch 12 Monate postoperativ (*p* = 0,04; Abb. 4). Auch nach Bereinigung um die Kovariaten „Alter bei Implantation“, „Dauer der Hörminderung“ und „Versorgungsmodus“ unterschied sich das Sprachverstehen hinsichtlich des Bildungsgrades sowohl 6 Monate als auch 12 Monate postoperativ statistisch signifikant (ANCOVA FBE 6 M: (*F* (2, 220) = 5,99; *p* < 0,01; partielles *η*^*2*^ = 0,05), ANCOVA FBE 12 M: (*F* (2, 220) = 4,05; *p* = 0,02; partielles *η*^*2*^ = 0,03)).

**Abb. 5:**
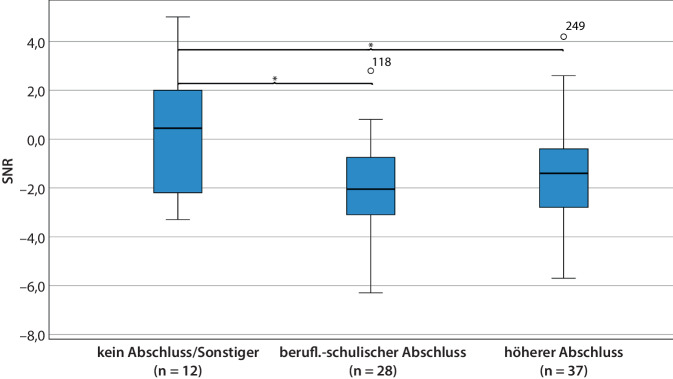
Boxplot OLSA 12 M post-OP im Zusammenhang mit dem Ausbildungsabschluss, *n* = 77, SNR („signal-to-noise ratio“), *p < 0,05; Oldenburger Satztest im Störgeräusch (OLSA, listenweiser Fallausschluss, Störgeräusch OlNoise, Sprachpegel 65 dB SPL fest, Störgeräuschpegel adaptiv, S0N0-Darbietung). *SNR* Signal-Rausch-Verhältnis („signal-to-noise ratio“)

Bei den Störgeräusch-Testergebnissen 12 Monate postoperativ zeigten sich gleichfalls signifikante Gruppenunterschiede (OLSA-Tests 12 M: *F* (2, 74) = 4,41; *p* = 0,02; *η*^*2*^ = 0,11). Unter Berücksichtigung der Kovariaten „Alter bei Implantation“, „Dauer der Hörminderung“ und „Versorgungsmodus“ war weiterhin für die Variable „Ausbildungsabschluss“ hinsichtlich des Sprachverstehens im Störgeräusch ein signifikanter Gruppenunterschied darstellbar (ANCOVA OLSA 12 M: *F* (2, 71) = 3,96; *p* = 0,02; partielles *η*^*2*^ = 0,10). Die Post-hoc-Analyse zeigte ein schlechteres Abschneiden der Fälle ohne Abschluss/mit sonstigem Abschluss im Vergleich zu der Gruppe mit beruflich-schulischem (*p* = 0,02) oder höherem Abschluss (*p* = 0,04; Abb. 5).

## Diskussion

### Zusammenfassung der Ergebnisse

Primäres Ziel der vorliegenden Studie war eine Untersuchung des Einflusses des Bildungsgrades auf die nach einer CI-Versorgung erreichte Hörleistung. Es zeigten sich signifikante Zusammenhänge zwischen dem Ausbildungsabschluss und den Ergebnissen sowohl in Ruhe als auch im Störgeräusch. Patienten mit einem beruflich-schulischen Abschluss erreichten signifikant schwächere Ergebnisse als solche mit einem höheren Abschluss, sowohl 6 Monate postoperativ (*p* < 0,01) als auch 12 Monate postoperativ (Freiburger Einsilbertest, *p* = 0,04; Abb. 4). Im Störgeräusch zeigte sich 12 Monate postoperativ ein schlechteres Abschneiden der Gruppe ohne Abschluss/sonstige Abschlüsse im Vergleich mit beruflich-schulischem (*p* = 0,02) oder höheren Abschluss (*p* = 0,04; Abb. 5). Die restlichen Angaben zum Bildungsgrad der Patienten, wie die Art der Betreuung im Kindesalter, die Schulform oder der Schulabschluss, zeigten keine signifikanten Zusammenhänge zum Sprachverstehen.

### Zusammenhang Bildungsgrad und Kognition

Der Bildungsgrad der Patienten fungierte in dieser Studie als möglicher Anhaltspunkt für deren kognitive Leistungsfähigkeit. In der Literatur sind die Einflüsse von Kognition auf das CI-Ergebnis zahlreich beschrieben. So zeigten mehrere Autoren, dass positive Korrelationen von verbaler Lernfähigkeit und dem auditiven Arbeitsgedächtnis mit dem Sprachverstehen bestehen [16, 20, 35]. Dieser Effekt ist ebenfalls bei CI-versorgten Kindern zu erkennen, wie von Pisoni beschrieben [34]. Auch die visuelle Gedächtnisleistung und die Fähigkeit, nonverbale Schlussfolgerungen zu treffen, korrelieren mit dem Zahlenverstehen, aber nicht mit gesprochenen Wörtern oder Sätzen [23]. Die vorliegende Studie zeigt eine schwache Korrelation zwischen dem Bildungsgrad als möglichem Indikator für die Kognition und dem Sprachverstehen bei CI-Patienten. Allerdings erklärt diese Variable nur wenige Prozentpunkte der Gesamtvarianz der Ergebnisse der Sprachverstehenstests. Unter Umständen ist der Bildungsgrad kein guter Anhaltspunkt zur Erfassung von individueller Intelligenz und Kognition, wie bereits von Heutink beschrieben. Heutink et al. gruppierten *n* = 129 Patienten basierend auf dem niederländischen Bildungssystems anhand ihres höchsten Bildungsabschlusses. Ein Abschluss eines Bachelor of Science (BSc) oder höher wurde als „hoher Bildungsgrad“ definiert und von *n* = 23 Studienteilnehmern erreicht. Der Mann–Whitney-Test zeigte im Rahmen der Auswertung keine Korrelation zwischen dem Bildungsgrad und den Ergebnissen des mit CI erreichten Sprachverstehens. Dieses Ergebnis ist möglicherweise auf die vorliegenden kleinen Gruppengrößen zurückzuführen. Die kleinste Gruppe in der statistischen Analyse der vorliegenden Studie umfasst *n* = 49 Fälle (kein Abschluss/Sonstiger), und ein hoher Bildungsgrad wurde von *n* = 115 Fällen erreicht. Dadurch ist eine bessere statistische Aussagekraft im Rahmen der Gruppenvergleiche gewährleistet. Signifikante Unterschiede sind potenziell erst bei Vorliegen von größeren Gruppen messbar. In einer weiteren Studie von Lazard et al. wurde der Bildungsgrad der Patienten anhand der Zeit in der Schule/Ausbildung definiert [25]. Die Autoren konnten keinen signifikanten Zusammenhang zwischen dem Bildungsgrad und dem Sprachverstehen der Patienten aufzeigen (*p* = 0,25). Bei Lazard et al. sind die Gruppen zwar deutlich größer als bei Heutink et al., allerdings erfolgt keine klare Abgrenzung nach der Art der Ausbildung, sondern es wurde lediglich die aufgewandte Zeit in besagtem Ausbildungsverhältnis berücksichtigt (Gruppe 1: < 12 Jahre, *n* = 21; Gruppe 2: 12–18 Jahre, *n* = 501; Gruppe 3: > 18 Jahre, *n* = 518, keine Angaben: *n* = 1211). Dadurch ist nur eine sehr arbiträre Einschätzung der Qualität des von den Patienten verfolgten Bildungsweges möglich, und die Gruppen erscheinen zu homogen, um statistisch relevante Unterschiede zu erfassen.

In der vorliegenden Studie ist eine Einteilung des Bildungsgrades hauptsächlich anhand der Schulform und des erreichten Abschlusses vorgenommen worden. Jedoch bestehen möglicherweise selbst innerhalb der gleichen Schulform regionale Unterschiede in Qualität und Quantität der Betreuung. Diese Differenzen sind jedoch nur sehr schwer messbar und können bei der betrachteten Stichprobe zu einer Verzerrung der Ergebnisse führen. Weiterhin ist der Bildungsgrad nur als potenzieller Indikator für die Kognition der Patienten anzusehen. Unter Umständen beeinflussen auch andere Faktoren den Bildungsweg der Patienten. Zum Beispiel ist die Priorisierung eines anderen Lebensstils losgelöst von der Weiterführung des Bildungsweges nicht automatisch mit einer geringeren Kognition gleichzusetzen. Soziale oder gesundheitliche Umstände können ebenfalls den weiteren Bildungsweg prägen und zu einem frühzeitigen Abschluss oder Abbruch der Schul- oder Hochschulbildung bzw. der Ausbildung führen. Genauere Aussagen sind nur durch die Anwendung kognitiver Testverfahren zu erwarten.

Ein Teil der in der vorliegenden Arbeit vorgestellten Daten wurde auszugsweise für einen Beitrag bei der 26. Jahrestagung der Deutschen Gesellschaft für Audiologie (DGA) analysiert [4]. Infolge einer leicht abweichenden Gruppeneinteilung der Variable „Schulform“ (Fälle der Schwerhörigen- und Gehörlosenschule gemeinsam gruppiert) konnten die Autoren zeigen, dass auch die Schulform neben dem Ausbildungsabschluss einen signifikanten Zusammenhang zu den Ergebnissen des Sprachverstehens mit CI aufweist. Dies bestärkt den in der vorliegenden Studie ermittelten statistischen Trend des Einflussfaktors „Schulform“ auf das Sprachverstehen. Die Daten der vorliegenden Studie deuten insgesamt darauf hin, dass Patienten mit einem höheren Bildungsgrad bessere Ergebnisse in den Sprachtests erzielen. Da der Bildungsgrad nur einen sehr kleinen Anteil der Gesamtvarianz der Ergebnisse der Sprachverstehenstests erklärt, ist die Bedeutung dieses Befundes möglicherweise gering.

### Einfluss von Hörgerätenutzung auf die sozioökonomische Position

Im Gegensatz zu den CI-Versorgungen ist der Einfluss des Bildungsgrades auf die Hörgerätenutzung in der Literatur zahlreich beschrieben [17, 31, 38]. Helvik et al. zeigt, dass bei *n* = 11.602 Patienten ≥ 65 Jahre die Hörgerätenutzung positiv mit einem höheren Bildungsgrad korreliert. Malcolm et al. beschreiben außerdem, dass nicht nur der Bildungsgrad, sondern die gesamte sozioökonomische Position (SEP) des Patienten mit der Nutzung von Hörgeräten zusammenhängt. Patienten mit einer niedrigeren SEP nutzen seltener Hörgeräte, sind häufiger arbeitslos und haben weniger Einkommen. Insgesamt zeigt sich deutlich eine positive Korrelation zwischen Hörgerätenutzung und dem Bildungsgrad. Jedoch ist der entsprechende Zusammenhang schwierig zu der vorliegenden Studie in Bezug zu setzen. Es wird nicht die Frage der Nutzung des CI untersucht, sondern der Erfolg der Versorgung (gemessen am Sprachverständnis der Patienten) in Bezug auf den Bildungsgrad.

### Alter bei Implantation

Das Alter bei Implantation ist, wie in vielen Studien gezeigt, ein gesicherter Einflussfaktor auf das Sprachverstehen nach CI-Versorgung [3, 10, 14, 29, 37]. Eine Überlagerung des Zusammenhangs von Kognition und Sprachverstehen ist ebenfalls in der Literatur beschrieben. So zeigten die Autoren in der Studie von Holden et al., dass nach Korrektur der Ergebnisse hinsichtlich des Alters keine signifikanten Zusammenhänge von Kognition und Sprachverstehen mehr bestanden [20]. Es ist gut dokumentiert, dass eine Veränderung des Hörvermögens mit zunehmendem Alter sowohl in den zentralen als auch in den peripheren Teilen des auditorischen Systems auftritt [13]. Ebenfalls als gesichert erscheint das altersbedingte Nachlassen kognitiver Fähigkeiten [37]. Beide Faktoren zusammen könnten für die beobachtete Verringerung der Hörleistung bei älteren CI-Patienten verantwortlich sein. Auch in der vorliegenden Studie ist eine negative Korrelation zwischen dem Alter bei Implantation und dem Sprachverstehen in Ruhe sowie eine positive Korrelation zwischen Alter und den Ergebnissen des Sprachverstehens im Störgeräusch festzustellen (12 Monate postoperativ). Allerding existieren auch Studien, die keinen altersbedingten Einfluss feststellten. Leung et al. zeigte, dass in der untersuchten Patientenkohorte ≥ 65 Jahre das Alter bei Implantation keinen signifikanten Einflussfaktor darstellte [28]. Bei gleichen Rahmenbedingungen (≥ 65 Jahre bei Implantation) zeigt sich auch in der vorliegenden Studie kein signifikanter Zusammenhang mit dem Sprachverstehen in Ruhe ((FBE 6 M: *r*_*S*_ (57) = −0,23; *p* = 0,08), (FBE 12 M: *r*_*S*_ (57) = −0,13; *p* = 0,32)) und im Störgeräusch ((OLSA 6 M: *r*_*S*_ (13) = −0,10; *p* = 0,75), (OLSA 12 M: *r*_*S*_ (13) = 0,12; *p* = 0,69)). Budenz et al. wiesen darauf hin, dass bei Berücksichtigung der Dauer der Hörminderung das Alter bei Implantation nicht mehr signifikant mit dem Sprachverstehen korrelierte [5]. Insgesamt bestätigen die Ergebnisse der vorliegenden Studie das Alter bei Implantation als sicheren Einflussfaktor auf das Sprachverstehen bei CI-Patienten, wenn eine breitere Altersverteilung zur Auswertung herangezogen wird.

### Dauer der Hörminderung

Die Dauer der Hörminderung ist der wohl stärkste und am besten dokumentierte Einflussfaktor auf das Sprachverstehen nach CI-Versorgungen. Je länger die Hörminderung besteht, desto schlechter scheint die CI-Performance hinsichtlich des Sprachverstehens zu sein [1, 3, 11, 20]. Vor allem die Dauer einer mindestens an Taubheit grenzenden Hörminderung („severe to profound hearing loss“, SPHL) ist ausschlaggebend. In der vorliegenden Studie waren keine Zusammenhänge zwischen der Dauer der Hörminderung und dem Sprachverstehen nach CI-Versorgung darstellbar. Dies könnte daran liegen, dass die Angaben bei der Erhebung der Eigenanamnese möglicherweise durch das nachlassende Erinnerungsvermögen vieler Patienten verzerrt vorliegen. Wegen der schwierigen Bestimmung des genauen Zeitpunkts des Beginns der Hörminderung erfolgte die Kategorisierung in sehr grob gefassten Gruppen (*Kongenital, Perilingual, Postlingual <* *5 Jahre, Postlingual 5–20 Jahre, Postlingual >* *20 Jahre*). Dadurch könnten bestehende Unterschiede verschleiert werden. Bernhard et al. zeigt in seinem systematischen Review von 36 Studien, dass insgesamt die Dauer der Hörminderung negativ mit dem Sprachverstehen der CI-Patienten korreliert [1]. Eine längere Dauer der Hörminderung resultiert in schlechteren Ergebnissen. Dieser Einflussfaktor verliert allerdings an Bedeutung bei zunehmender CI-Erfahrung. Die Dauer des Hörverlusts konnte in der vorliegenden Studie nicht signifikant mit dem Sprachverstehen der Patienten in Verbindung gebracht werden, obwohl sie in der Literatur bisher als Einflussfaktor beschrieben wurde.

### Versorgungsmodus

Im Hinblick auf das Sprachverstehen in Ruhe (FBE) deuten die Ergebnisse dieser Studie auf ein schlechteres Sprachverstehen der SSD-versorgten Patienten hin. Das Sprachverstehen im Störgeräusch (OLSA) führt zu disparaten Resultaten. Diese Unterschiede hängen möglicherweise mit der Art der Vertäubung der Patienten zusammen. Im Rahmen des OLSA-Tests wurde bei Probanden mit einseitiger Ertaubung und nutzbarer Hörfunktion des Gegenohres das kontralaterale Ohr mittels Ohrstöpseln und Kapselgehörschutz (Peltor) doppelt abgeschirmt. Diese Art der Vertäubung gewährleistet unter Umständen keinen vollständigen Ausschluss des nicht getesteten Ohres. Deshalb erzielten SSD-versorgte Patienten auch die besten Ergebnisse, da bei ihnen eine Normakusis auf der kontralateralen Seite vorlag und eine ausreichende Vertäubung oder Maskierung möglicherweise nicht in allen Fällen möglich war. Eine weitere Unsicherheit kommt dadurch zustande, dass die Bestimmung des Modus präoperativ anhand des mit Hörgeräte-Versorgung im Freifeld aufgezeichneten Sprachaudiogramms erfolgte. Möglicherweise lag in einigen Fällen eine Änderung des Versorgungsmodus vor, ohne dass dieser in der Datenbank begleitend erfasst wurde. Somit sind weitere Untersuchungen notwendig, um den Einflussfaktor „Versorgungsmodus“ belegen zu können.

### Limitationen der Studie

Neben den bereits im Diskussionsteil dargestellten Limitationen gibt es potenziell weitere Faktoren, welche die Ergebnisse der Studie beeinflussen: Die Angaben zur Hörbiografie erfolgten in der Regel durch eine Eigenanamneseerhebung. Besonders bei betagten Patienten könnten aufgrund nachlassender Erinnerungsfähigkeit fehlerhafte Angaben zum Schulsystem aufgetreten sein. Weiterhin erschwert das Vorliegen von vielen verschiedenen Implantatsystemen, Elektrodenbauformen und CI-Prozessoren die Vergleichbarkeit der Testergebnisse, da Unterschiede in der Technologie möglicherweise die Hörleistungen beeinflussen. Die Nachkontrollen vom FBE und OLSA erfolgten 6 und 12 Monate postoperativ. Es wurde ein relativ breites Zeitfenster von ± 4 Monaten zur Auswertung angesetzt, wodurch möglicherweise eine Vergleichbarkeit der Patientenverläufe erschwert ist. In der vorliegenden Studie wurden mehrere andere Einflussfaktoren betrachtet, welche teilweise einen signifikanten Einfluss auf das Sprachverstehen aufwiesen (Alter bei Implantation, Versorgungsmodus). Hinsichtlich dieser Kovariaten wurden die Ergebnisse korrigiert, allerdings könnten noch weitere nicht bestimmte Kovariaten den Einfluss des Bildungsgrades auf das Sprachverstehen überdecken.

### Zusammenfassung

Die Daten dieser Studie konnten wichtige, bereits bekannte Einflussfaktoren auf das CI-Ergebnis bestätigen. Insgesamt deuten die Ergebnisse darauf hin, dass der Bildungsgrad der Patienten einen Einfluss auf das Sprachverstehen nach CI-Versorgung haben könnte. Eine genauere Unterteilung der verschiedenen Bildungsgrade mit größeren Datensätzen könnte helfen, diesen Zusammenhang zu bestätigen.

## Fazit für die Praxis

- Der Bildungsgrad zeigt eine schwache Korrelation zum Sprachverstehen nach Cochleaimplantat(CI)-Versorgung auf.
- Ein höherer Bildungsgrad scheint sich leicht positiv auf das Sprachverstehen auszuwirken.
- Ein großer Teil der Varianz des Sprachverstehens nach CI-Versorgung bleibt weiterhin ungeklärt.

## Supplementary Information

Formular „Arbeit und Leben“, Datenbank ENT-Statistics, Fa. Innoforce, Liechtenstein

## Data Availability

The data that support the findings of this study are not openly available due to reasons of sensitivity and are available from the corresponding author upon reasonable request. Data are located in controlled access data storage at Goethe University Frankfurt, Germany.
