# Supplementary material for: Factors influencing the outcome of cochlear implantation: what role is played by secondary and post-secondary education? German version
Source: HNO. 2025 Jul 31;73(9):650–60. [Article in German] doi: 10.1007/s00106-025-01646-9 (PMC12370789; doi:10.1007/s00106-025-01646-9)
Supplement: Supplementary file 1 — Formular „Arbeit und Leben“, Datenbank ENT-Statistics, Fa. Innoforce, Liechtenstein [file 106_2025_1646_MOESM1_ESM.pdf]

**Universitätsklinikum Frankfurt**
Theodor-Stern-Kai 7
60590 Frankfurt am Main
Deutschland

# Arbeit & Leben vom 20.01.2025

BOND James, 02.03.1968 (56 j), m — Alter am 20.01.2025

| | |
|---|---|
| PID (FID): | 7 (1111) |
| Datum: | 20.01.2025 |

## Wohnsituation

**Familie**

## Umfeld

**Hörend**

## Lautsprache

**Verständlich, aber deutliche Sprachstörung**

## Frühkindliche/schulische Laufbahn

**Kindergarten**

↳ **Regelkindergarten**

**Schule**

↳ **Regelschule**

## Schulabschluss (höchster)

**Realschulabschluss (Mittlere Reife)**

## Ausbildungsabschluss (höchster)

**Meister-, Technikerschule, Berufs-/ Fachakademie**

## Tätigkeit

**Arbeitend**

## Ausbildungsabschluss (höchster)

- ○ keinen berufl. Abschluss
- ○ berufl.-schulische Ausbildung (Handelsschule/Berufsfachschule)
- ◉ Meister-, Technikerschule, Berufs-/ Fachakademie
- ○ Fachhochschulabschluss
- ○ Hochschulabschluss
- ○ anderer beruflicher Abschluss

**Kindergarten**

- (•) Regelkindergarten
- ( ) Integrationskindergarten
- ( ) Schwerhörigenkindergarten
- ( ) Gehörlosenkindergarten
- ( ) Kein

## Schulabschluss (höchster)

- ( ) ohne Schulabschluss
- ( ) Hauptschulabschluss
- (•) Realschulabschluss (Mittlere Reife)
- ( ) Fachhochschulreife
- ( ) Hochschulreife (allg./fachgeb.)
- ( ) Polytechn. Oberschule 10. Klasse
- ( ) Anderer Schulabschluss

**Schule**

- (●) Regelschule
- ( ) Integrationsschule
- ( ) Schule für Lernhilfe
- ( ) Schwerhörigenschule
- ( ) Gehörlosenschule
- ( ) Keine
- ( ) Sonstiges

## Tätigkeit

- [ ] In Schulausbildung (allgemeinbildend)
- [ ] In Berufs- (Hochschul) -ausbildung
- [x] Arbeitend
- [ ] Ruhestand/Pension
- [ ] ohne Beschäftigung / arbeitsuchend
